# Early-life gut dysbiosis programs divergent metabolic trajectories in a sex-dependent manner

**DOI:** 10.1080/19490976.2026.2718681

**Published:** 2026-08-20

**Authors:** Junlei Su, Yunchong Guo, Lijun Shen, Yaqing Wen, Bihui Huang, Fangping Li

**Affiliations:** a Department of Endocrinology, The Seventh Affiliated Hospital, Sun Yat-sen University, Shenzhen, China; b The Shenshan Medical Center, Sun Yat-sen Memorial Hospital, Sun Yat-sen University, Shenzhen, China

**Keywords:** Gut dysbiosis, sex dimorphism, adiposity, hepatic steatosis, antibiotics

## Abstract

Previous studies have established that long-term antibiotic (ABX) exposure disrupts gut microbiota, leading to adiposity, insulin resistance, and metabolic dysfunction-associated steatotic liver disease (MASLD). However, the metabolic effects of short-term ABX treatment in early life, along with the underlying mechanism, remain unclear. This study reveals that short-term ABX exposure during early life induces sex-specific metabolic programming in mice. Immature males exhibited reduced gonadal white adipose tissue (gWAT) mass, predisposing them to exacerbated hepatic steatosis under high-fat diet (HFD) challenge. Females displayed protected liver metabolism. We identified dimethylarsinous acid (DMAIII) as a key microbiota-dependent metabolite that inversely correlated with both gWAT mass and specific bacterial abundances, including *Muribaculum gordoncarteri* and *Ligilactobacillus apodemi* in male mice. Critically, timely fecal microbiota transplantation (FMT) reversed these metabolic alterations in males. Our findings demonstrate that transient early-life microbiota disruption programs lasting, sex-divergent metabolic outcomes, highlighting the therapeutic potential of microbiota-targeted interventions during developmental windows.

## Graphical abstract

**Figure:**
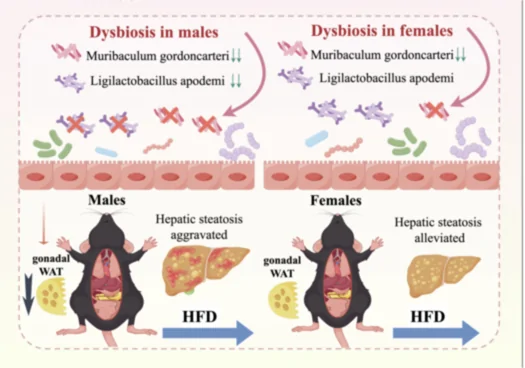

## 1. Introduction

During the initial weeks of life, colonization by facultative anaerobes reduces oxygen levels, facilitating the proliferation of strict anaerobes. The gut microbiota undergoes dynamic compositional shifts in response to dietary changes and host development throughout the first year, culminating in dominance by *Bacteroides* and *Firmicutes* by 12 months of age. Further maturation occurs over the next 2 years, with the infant microbiome gradually stabilizing and reaching an adult-like composition by age three.^1^ The gut microbiota dynamically adapts to environmental exposures and host development, playing a crucial role in maintaining intestinal homeostasis.

Antibiotics (ABX) are among the most frequently prescribed medications for children, but inappropriate use can lead to detrimental health consequences. Longitudinal studies have revealed that ABX exposure exerts significant short- and long-term effects on the diversity and composition of the gut microbiota.^2^ Abrupt gut disruptions caused by ABX not only contribute to adverse drug reactions and accelerate the development of antibiotic resistance but also play a causal role in the pathogenesis of various diseases.^3^ Growing evidence suggests that gut dysbiosis is associated with several non-communicable diseases such as asthma, allergy, and obesity. These diseases are increasing in prevalence in many regions of the world.^4^

Because early life is a critical period for metabolic development, farmers use low doses of ABX to promote livestock accumulating fat content. In humans, it has been reported that early-life microbiota disruption induced by ABX is associated with increased risk of overweight status later in childhood^5^ with a sex-specific effect, especially in boys.^6^ However, the underlying mechanism remains to be revealed. Researchers have applied the agricultural model of growth promotion to explore how ABX exposure modulates host metabolic phenotypes. They gave subtherapeutic antibiotic therapy (STAT) to young mice for more than 7 weeks. It was observed that metabolic hormones were altered, fat mass was increased, hepatic metabolism of lipids and cholesterol was strengthened, and colonic short-chain fatty acid (SCFA) levels were increased, which could stimulate adipogenesis.^7^ Cox, L.M. et al. found that early-life low-dose penicillin (LDP) exposure in mice for 4–8 weeks induced persistent changes in body composition, resulting in increased total, lean, and fat mass compared to controls. This suggests that infant microbiota interactions critically influence long-term host metabolism. Moreover, the growth-promoting phenotype was transferable to germ-free mice via LDP-shaped microbiota, confirming a causal role of the altered microbiota rather than the antibiotic itself.^8^

Our investigation into the long-term metabolic consequences of short-course ABX exposure revealed a profound sexual dimorphism. In immature males, ABX treatment led to a specific reduction in gonadal white adipose tissue (gWAT) mass, an effect absent in females. This phenotype was mechanistically linked to a concomitant exacerbation of high-fat diet-induced hepatic steatosis, implicating a microbiota-dependent partitioning of lipid storage. The capacity of fecal microbiota transplantation (FMT) to reverse both the adipose and hepatic phenotypes underscores a causal role for the gut microbiome. Intriguingly, early-life ABX exerted a protective effect against hepatic steatosis in females, highlighting a fundamental sex-specificity in the developmental programming of metabolism by the microbiome.

## 2. Materials and methods

### 2.1. Mice

Specific pathogen-free (SPF) male and female C57BL/6 mice (Guangdong Medical Lab Animal Center) were housed under controlled conditions (12-h light/dark cycle, 22–24 °C, 50%–70% humidity) with free access to food and water. Starting at 3 weeks of age, mice were maintained on a standard laboratory chow diet, then in the high-fat diet (HFD) challenge experiment, they were transitioned to an HFD at 9 weeks of age. Same-sex littermates were randomly distributed into experimental groups. Food consumption was monitored per cage over 72-hour periods and normalized to average daily intake per mouse. Following a 6-hour fast, all animals were euthanized. All procedures were conducted in accordance with the Institutional Animal Care and Use Committee of Sun Yat-Sen University (SYSU-IACUC-2024-001362) and conformed to relevant ethical guidelines for animal research.

### 2.2. Diet

The standard chow diet was a standard maintenance diet for mice, obtained from the Guangdong Medical Laboratory Animal Center. It provides an energy content of 3.5 kcal/g, with crude protein constituting 185 g/kg, accounting for 21% of the total energy, and a crude fiber content of 32 g/kg. The high-fat diet (HFD) was D12492i (60% kcal fat), sourced from Research Diets. This diet has an energy density of 5.21 kcal/g, with crude protein contributing 20% of the total energy (derived from lard at 245 g/kg and soybean oil at 25 g/kg), and a crude fiber content of 50 g/kg.

### 2.3. Antibiotics (ABX) treatment

The antibiotic (ABX) cocktail, consisting of vancomycin hydrochloride (125 mg/L; Meilunbio) and gentamicin sulfate (100 mg/L; Meilunbio), was administered in drinking water to mice starting at 4 weeks of age for a duration of 2 weeks. The ABX solution was replaced weekly to ensure stability. Control mice received ABX-free drinking water ad libitum.

### 2.4. The ABX-induced gut microbiota dysbiosis model

Male and female immature mice were used to establish a model of gut microbiota dysbiosis using ABX treatment. At 4 weeks of age, vancomycin and gentamicin were added to the drinking water, and the mice were allowed free access for 2 weeks. A control group consisted of 4-week-old immature mice that received ABX-free drinking water ad libitum. In this model, each group contained 5 mice. One mouse in the ABX-treated male (M6W-ABX) group was excluded from untargeted metabolite profiling and serum TG detection due to insufficient serum.

### 2.5. The fecal microbiota transplantation (FMT) model

Four-week-old C57BL/6 recipient mice were pretreated with an ABX cocktail for 2 weeks, with treatment discontinued 24 h prior to FMT. Fresh feces were collected from sex- and age-matched conventionally raised donor mice, which were co-housed by sex. Fecal slurry was prepared immediately in sterile PBS at a concentration of 125  mg/mL and administered to recipients via oral gavage at a volume of 100 μL, three times per week. In the FMT model experiments, two independent animal experiments were conducted. The first experiment was performed to monitor changes in body weight and food intake of the mice; each group contained 5 mice, but one mouse in the male control group (Control-M) and one mouse in the male PBS-gavage group (PBS-M) were dead during gavage.

The second experiment was designated for adipose tissue and serum sample collection. In this experiment, both the male (Control-M) and female control groups (Control-F) comprised 4 mice, and the remaining 4 groups comprised 5 mice each. One mouse in the Control-M group was excluded from untargeted metabolite profiling, serum ALT, and AST detection due to insufficient serum. One mouse in the Control-M group was excluded from the relative Col1a1 mRNA expression in gWAT due to an insufficient gWAT sample.

**The control group** (Control-M and Control-F): Four-week-old male or female immature mice receive ABX-free drinking water for 2 weeks. At 6 weeks of age, they were orally gavaged with 100 µL of anaerobic, sterile PBS for 3 weeks.

**The PBS group** (PBS-M and PBS-F): Four-week-old male or female immature mice were given ABX-containing drinking water for 2 weeks, followed by oral gavage with 100 µL of anaerobic, sterile PBS for 3 weeks starting at 6 weeks of age.

**The experimental group** (MGM and FGF): Four-week-old male or female immature mice received ABX-containing drinking water for 2 weeks as described above. At 6 weeks of age, the ABX were withdrawn, and the mice were administered 100 µL of fecal slurry by oral gavage for 3 weeks.

### 2.6. The short-term HFD model

The methods for establishing gut microbiota dysbiosis and performing FMT in immature mice were as described above. All experiments were conducted separately by sex. In this model, each group contained 5 mice, one mouse in the male control group (Control-M-HFD) and one mouse in the male PBS-gavage group (PBS-M-HFD) died during gavage. One mouse in Control-M-HFD group was excluded from untargeted metabolite profiling due to insufficient serum. One mouse in MGM-HFD group was excluded from fecal 16S rRNA gene sequencing analysis due to an insufficient fecal sample.

**The HFD control group** (Control-M-HFD and Control-F-HFD): Mice received no ABX treatment during the juvenile stage. Starting at 6 weeks of age, they were orally gavaged with anaerobic, sterile PBS for 3 weeks. From 9 weeks of age, they were fed HFD for 4 weeks.

**The HFD PBS group** (PBS-M-HFD and PBS-F-HFD): At 4 weeks of age, mice were treated with ABX for 2 weeks. From 6 weeks of age, they received oral gavage with anaerobic, sterile PBS for 3 weeks. From 9 weeks of age, they were fed HFD for 4 weeks.

**The HFD experimental group** (MGM-HFD and FGF-HFD): At 4 weeks of age, mice were treated with ABX for 2 weeks. From 6 weeks of age, they received oral gavage with fecal slurry from sex-matched donors for 3 weeks. From 9 weeks of age, they were fed HFD for 4 weeks.

### 2.7. Glucose tolerance test (GTT)

Following a 14-hour fast with free access to water, mice received an intraperitoneal injection of glucose (1 g/kg). Blood glucose levels were measured from the tail vein immediately before (0 min) and at 15, 30, 60, 90, and 120 min after injection using a commercial glucometer and test strips (Yuyue, China).

### 2.8. TG and TC measurements

Following a 6-hour fast, mice were euthanized for serum collection. Serum lipids (TG and TC) were quantified using commercial kits (Kehua, China). Hepatic TG content was determined from liver homogenates using a corresponding assay kit (Beyotime, China). All procedures were strictly followed according to the manufacturer's instructions.

### 2.9. Oil red O staining

Liver tissue samples were fixed in 4% paraformaldehyde (12–24 h, 4 °C), cryoprotected by equilibration in 15% and 30% sucrose (overnight each at 4 °C), and embedded in OCT compound. Cryosections (8–10 μm) were affixed to slides, air-dried, and post-fixed in 4% PFA. For lipid staining, sections were incubated in filtered Oil Red O working solution (8–10 min, protected from light), differentiated in 75% ethanol, and counterstained with Mayer’s hematoxylin. After differentiation in acid-alcohol and bluing in ammonia water, sections were mounted with aqueous glycerol gelatin.

### 2.10. Hematoxylin and eosin (H&E) staining

Liver and adipose tissue samples were fixed for 24 h, processed through graded ethanol (75%–100%), cleared in xylene, and infiltrated with paraffin wax using an automated tissue processor. Following embedding, 4-μm sections were cut, floated on a 40 °C water bath, and mounted on slides. For H&E staining, sections were deparaffinized, rehydrated, and stained with hematoxylin (3–5 min) with differentiation in acid-alcohol and bluing in ammonia water. Cytoplasmic staining was performed with eosin (5 min), followed by dehydration, clearing in xylene, and mounting with neutral balsam.

### 2.11. Masson staining

Paraffin sections of liver tissue were deparaffinized and rehydrated through sequential immersion in xylene (2 × 20 min), absolute ethanol (2 × 5 min), and 75% ethanol (5 min), followed by a tap water rinse. Sections were then incubated overnight in potassium dichromate solution, briefly washed, and stained with iron hematoxylin (1:1 mixture of solutions A and B, 3 min). After differentiation in acid-alcohol and rinsing, sections were stained with Ponceau Acid Fuchsin (5–10 min), treated with phosphomolybdic acid (1–3 min), and directly transferred to aniline blue staining solution (3–6 min). Following brief differentiation in 1% acetic acid, sections were dehydrated in absolute ethanol, cleared in *n*-butanol and xylene, air-dried, and mounted with neutral balsam.

### 2.12. Gut microbiota DNA extraction and 16S rRNA gene sequencing

#### 2.12.1. DNA extraction

Genomic DNA was extracted from fecal and cecal content samples using the MagPure Stool DNA KF Kit B (MAGEN, China) according to the manufacturer's protocol. Briefly, 100–200 mg of sample was homogenized with grinding beads in 1 mL of Buffer ATL/PVP-10 using a tissue homogenizer (Jingxin Tech, China), followed by incubation at 65 °C for 20  min. After centrifugation at 14,000 × g for 5 min, the supernatant was collected and mixed with 0.6 mL of Buffer PCI by vortexing for 15 s. The mixture was centrifuged at 18,213 × g for 10 min, and the resulting supernatant was transferred to a deep-well plate pre-loaded with magnetic bead binding solution. Automated nucleic acid purification was performed on a KingFisher Flex system (Thermo Fisher, USA). Eluted DNA was stored at −80 °C until further processing.

#### 2.12.2. Sequencing data processing

16S rRNA gene sequencing was performed on the DNBSEQ-G400 platform (BGI, Shenzhen, China), a high-throughput sequencing system based on DNBSEQ™ technology. This platform utilizes DNA nanoball (DNB) technology and combinatorial probe anchor synthesis (cPAS), which enables high accuracy, low duplication rates, and minimal index hopping. Paired-end 300 bp (PE300) sequencing was employed to obtain high-quality reads covering the variable regions of the 16S rRNA gene. Raw sequencing data were quality-filtered to generate high-quality clean reads using the following criteria: (1) reads were trimmed using a sliding window (25 bp) when the average quality score dropped below 20, and those shorter than 75% of their original length were discarded; (2) reads containing adapter sequences (allowing ≤3 mismatches over a 15 bp overlap) were removed; (3) reads with ambiguous bases (N) were excluded; and (4) low-complexity reads containing homopolymers ≥10 bp were filtered out. Demultiplexing was performed, allowing zero mismatches in barcode sequences.

#### 2.12.3. OTU clustering, taxonomic annotation, and statistical analysis

Paired-end reads were merged into tags using FLASH (v1.2.11) with a minimum overlap of 15 bp and a maximum mismatch rate of 0.1. Tags were clustered into operational taxonomic units (OTUs) at 97% similarity using USEARCH (v7.0.1090) via the UPARSE algorithm. Chimeric sequences were detected and removed with UCHIME (v4.2.40) against the Greengenes database for 16S rRNA data. All tags were mapped back to non-chimeric OTU representatives to generate an OTU abundance table. Taxonomic assignment was performed by comparing representative sequences to the Greengenes database. Based on the OTU table and taxonomic annotations, alpha diversity (within-sample diversity) and beta diversity (between-sample compositional differences) were calculated. Subsequent association analyses were performed to evaluate group-wise microbial community variations.

### 2.13. Untargeted metabolomics

#### 2.13.1. Sample preparation

Serum samples were thawed at 4 °C and aliquoted (100  μL) into new tubes. Metabolites were extracted by adding 700  μL of pre-cooled extraction solvent (methanol:acetonitrile:water = 4:2:1, v/v/v) containing internal standards (d3-Leucine, 13C9-Phenylalanine, d5-Tryptophan, and 13C3-Progesterone). After vortexing for 1 min, samples were incubated at −20 °C for 2 h, followed by centrifugation at 25,000 × g for 15 min at 4 °C. The supernatant (600  μL) was collected and dried under vacuum. The residue was reconstituted in 180  μL methanol:water (1:1, v/v), vortexed for 10 min, and centrifuged again under the same conditions. The final supernatant was collected for LC–MS/MS analysis. Quality control (QC) samples were prepared by pooling equal aliquots (20  μL) from each sample.

#### 2.13.2. LC–MS/MS analysis

LC–MS/MS analysis was performed on a Q Exactive mass spectrometer (Thermo Scientific) equipped with an electrospray ionization source. Full MS scans were acquired at a resolution of 70,000 over m/z 70–1050, with an AGC target of 3e6 and maximum injection time of 100 ms. The top 3 precursors were selected for fragmentation with stepped normalized collision energies of 20, 40, and 60 eV. MS/MS spectra were acquired at a resolution of 17,500 with an AGC target of 1e5 and maximum injection time of 50 ms. ESI parameters were set as follows: sheath gas flow rate, 40; auxiliary gas flow rate, 10; spray voltage, 3.80 kV (positive mode) or 3.20 kV (negative mode); capillary temperature, 320 °C; auxiliary gas heater temperature, 350 °C.

#### 2.13.3. Data processing and metabolite identification

Raw data were processed using Compound Discoverer 3.3. (Thermo Scientific) with the following parameters: parent mass tolerance <5 ppm, fragment mass tolerance <10 ppm, and retention time deviation < 0.2 min. Metabolite identification was performed by querying the BGI Metabolome Database (BMDB), mzCloud, and ChemSpider. Data preprocessing was conducted using MetaX, including probabilistic quotient normalization (PQN), quality control-based robust LOESS signal correction (QC-RLSC), and removal of metabolites with a coefficient of variation >30% in QC samples.

#### 2.13.4. Statistical analysis

Principal component analysis (PCA) was performed after log-transformation and Pareto-scaling to evaluate data quality and visualize group separation. Clustering of QC samples in PCA score plots indicated satisfactory instrumental stability and data reproducibility.

### 2.14. Quantitative real-time PCR (qRT-PCR) analysis

Total white adipose tissue RNA was extracted using a commercial kit (ESscience, PC001) and reverse-transcribed into cDNA with the PrimeScript RT Reagent Kit (TAKARA, RR036A), according to the manufacturer's protocols. Quantitative PCR was performed using Green® Premix Ex Taq™ II (TAKARA, RR820A) on a Roche LightCycler 480II system. The Col1a1 gene was amplified using the primers: forward 5′-GCTCCTCTTAGGGGCCACT-3′, reverse 5′-CCACGTCTCACCATTGGGG-3′. The 36b4 gene (forward 5′-AGATTCGGGATATGCTGTTGGC-3′, reverse 5′-TCGGGTCCTAGACCAGTGTTC-3′) served as an internal control. Relative gene expression was calculated using the 2^–ΔΔCt method.

### 2.15. Western blotting analysis

Liver, brown adipose tissue, and gonadal white adipose tissue samples were homogenized in RIPA buffer and lysed on ice for 30 min. Following centrifugation at 21,130 × g for 30  min at 4 °C, supernatants were collected, and protein concentrations were determined using a BCA assay kit (Thermo Scientific). Proteins (30–50 μg) were separated by SDS–PAGE on 10% gels and transferred to nitrocellulose membranes. After blocking with 5% skim milk in TBST for 2 h at room temperature, membranes were incubated overnight at 4 °C with the following primary antibodies (all 1:1000): phospho-Akt (Ser473) (Abmart T55561), phospho-Akt (Thr308) (CST 9275S), Akt1/2/3 (Abmart T55561), Hsp90 (CST 4874S), and Ucp1 (Abcam ab10983). Membranes were then incubated with HRP-conjugated anti-rabbit secondary antibody (Beyotime, 1:3000) for 1 h at room temperature. Protein bands were visualized using enhanced chemiluminescence substrate (Meilunbio) and quantified with ImageJ software (NIH).

### 2.16. Data analysis and visualization

Data are presented as mean ± SD or median (interquartile range) based on data distribution characteristics. For comparisons between two groups, a two-sided independent samples *t*-test was employed, while one-way ANOVA with least significant difference (LSD) post hoc testing was applied for multi-group comparisons. Western blot quantification analyses followed the same statistical approaches. Statistical significance was defined as *p* < 0.05. All analyses were performed using SPSS 22.0 (SPSS Inc.). Analytical methods for bulk RNA sequencing and 16S rRNA gene sequencing data are described in the preceding sections.

In the aforementioned methodology section, the specific numbers of experimental mice in different experimental models are described in detail. The Venn diagram, correlation coefficient plot, and scatter plot in Figures 4a, b, e–k and 5a, b, d, e, h–m were plotted by https://www.bioinformatics.com.cn (last accessed on May 4, 2026), an online platform for data analysis and visualization.

## 3. Results

### 3.1. Immature mice have sex-dimorphic gut microbiota

We characterized the gut microbiota composition in male and female mice fed a standard chow diet across key developmental stages-weaning (3 weeks), pre-puberty (4 weeks), post-puberty (6 weeks), and young adulthood (8 weeks) by using 16S rRNA gene sequencing of fecal DNA samples (Figure S1a, b). Alpha diversity remained comparable between sexes in earlier developmental periods and young adulthood (Figure S1c–f). Beta diversity also remained comparable between sexes in earlier developmental periods but diverged significantly by young adulthood (Figure S1g–j). At the phylum level, *Bacteroidota* and *Firmicutes* dominated the microbial community throughout early life (Figure S1k). The relative abundance of *Ligilactobacillus,* a lactose-metabolizing genus implicated in energy homeostasis, varied dynamically with age, reaching its peak during pre-puberty (Figure S1l). Sex-specific microbiota profiles were further delineated using partial least squares-discriminant analysis (PLS-DA), which revealed distinct clustering patterns between sexes across developmental stages (Figure S1m–p).

### 3.2. Short-term antibiotic exposure induces gut dysbiosis and reduces adipose mass in immature male mice

**Figure 1.**
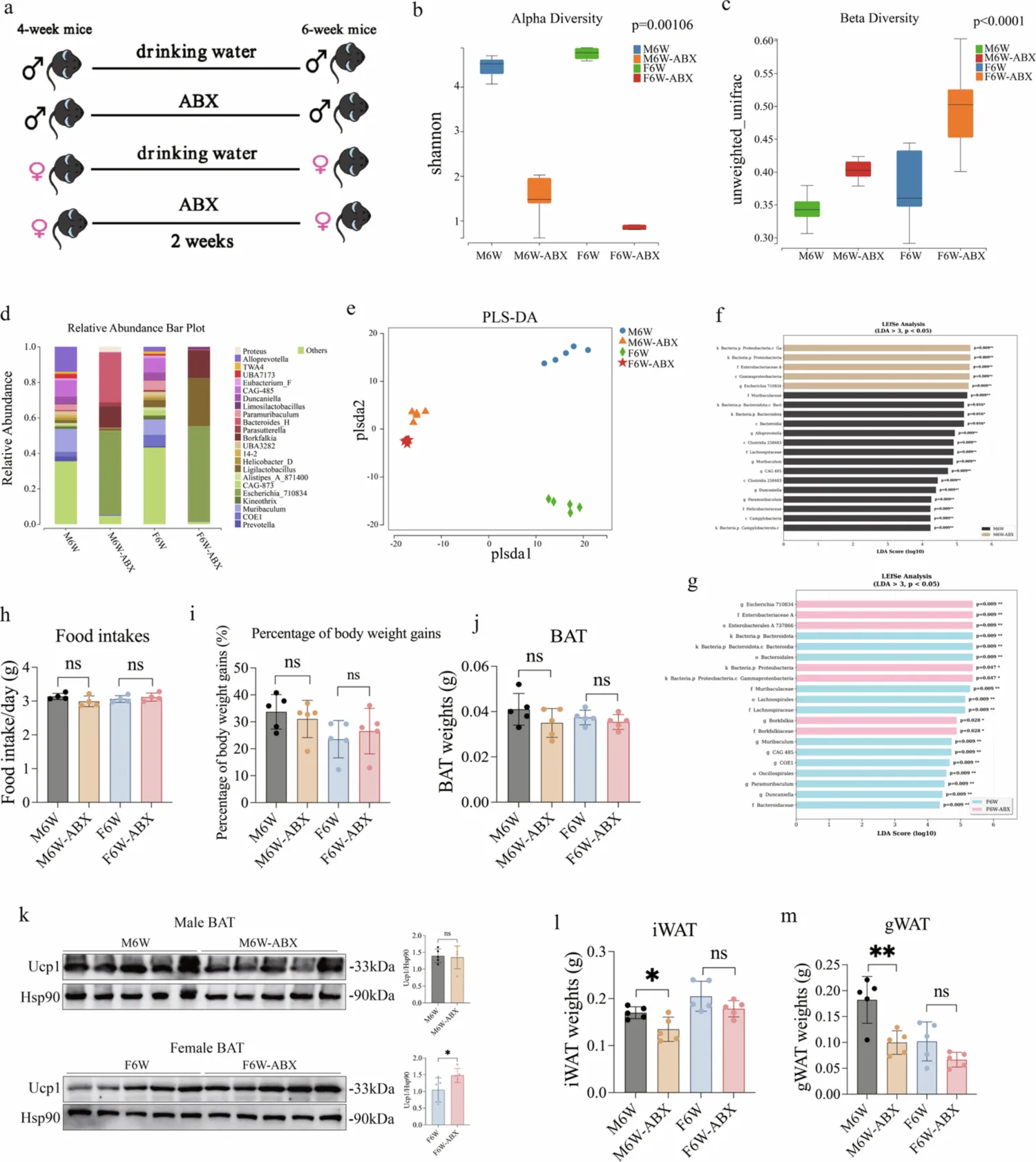
ABX treatment has sex-dimorphic effects on BAT and WAT. (a) Study design of 2-week ABX treatment. (b) Alpha diversity of fecal-derived gut microbiota. (c) Beta diversity of fecal-derived gut microbiota. (d) Community bar plot analysis of fecal-derived gut microbiota at the phylum level. (e) Partial least squares discriminant analysis (PLS-DA) plot of fecal-derived gut microbiota. (f) Linear discriminant analysis effect size (LEfSe) analysis of gut microbiota composition between M6W and M6W-ABX groups. (g) Linear discriminant analysis effect size (LEfSe) analysis of gut microbiota composition between F6W and F6W-ABX groups. (h) Food intakes, (i) percentage of body weight gains, (j) BAT weights, (k) Ucp1 and Hsp90 protein expression of BAT, (l) iWAT weights, and (m) gWAT weights were compared be between groups in both sexes. For b and c, values are expressed as the median (interquartile ranges). For f and g, LEfSe is performed to identify significantly discriminative taxa with a logarithmic LDA score threshold of > 3.0 and a significance level of *p* < 0.05. For h–m, values are expressed as the mean ± sem. *, *p* < 0.05; **, *p* < 0.01; ***, *p* < 0.001; ns, not significant. Abbreviation: ABX, antibiotics; M6W, ABX-naïve male mice; F6W, ABX-naïve female mice; M6W-ABX, ABX-treated male mice; F6W-ABX, ABX-treated female mice; BAT, brown adipose tissue; iWAT, inguinal white adipose tissue; gWAT, gonadal white adipose tissue.

To assess the impact of short-term ABX exposure on metabolism, prepubertal (4-week-old) mice on a standard chow diet were treated with an ABX cocktail for 2 weeks in males (M6W-ABX) and females (F6W-ABX) (Figure 1a). 16S rRNA sequencing of both fecal-derived (Figure 1b, c) and cecal-derived gut microbiota (Figure S2a, b) confirmed that ABX administration reduced alpha diversity while increasing beta diversity in both sexes. Taxonomic profiling at the genus level, together with PLS-DA and linear discriminant analysis effect size (LEfSe) analysis, demonstrated that ABX-induced gut microbiota alterations were distinct between males and females in both feces (Figure 1d–g) and cecal content (Figure S2c).

ABX treatment did not affect cumulative food intake, percentage of body weight gain, or brown adipose tissue (BAT) mass in either sex (Figure 1h–j). Although Ucp1 expression in BAT was unaltered in ABX-exposed males, a moderate upregulation was observed in females (Figure 1k). In contrast, ABX exposure selectively reduced both inguinal and gonadal white adipose tissue (iWAT and gWAT) mass in male mice, with no significant effect in females (Figure 1l, m). Serum levels of triglycerides (TG) and total cholesterol (TC) remained unchanged following ABX treatment in both sexes (Figure S3a, b).

### 3.3. FMT ameliorates gut dysbiosis-induced gWAT mass loss in immature male mice

**Figure 2.**
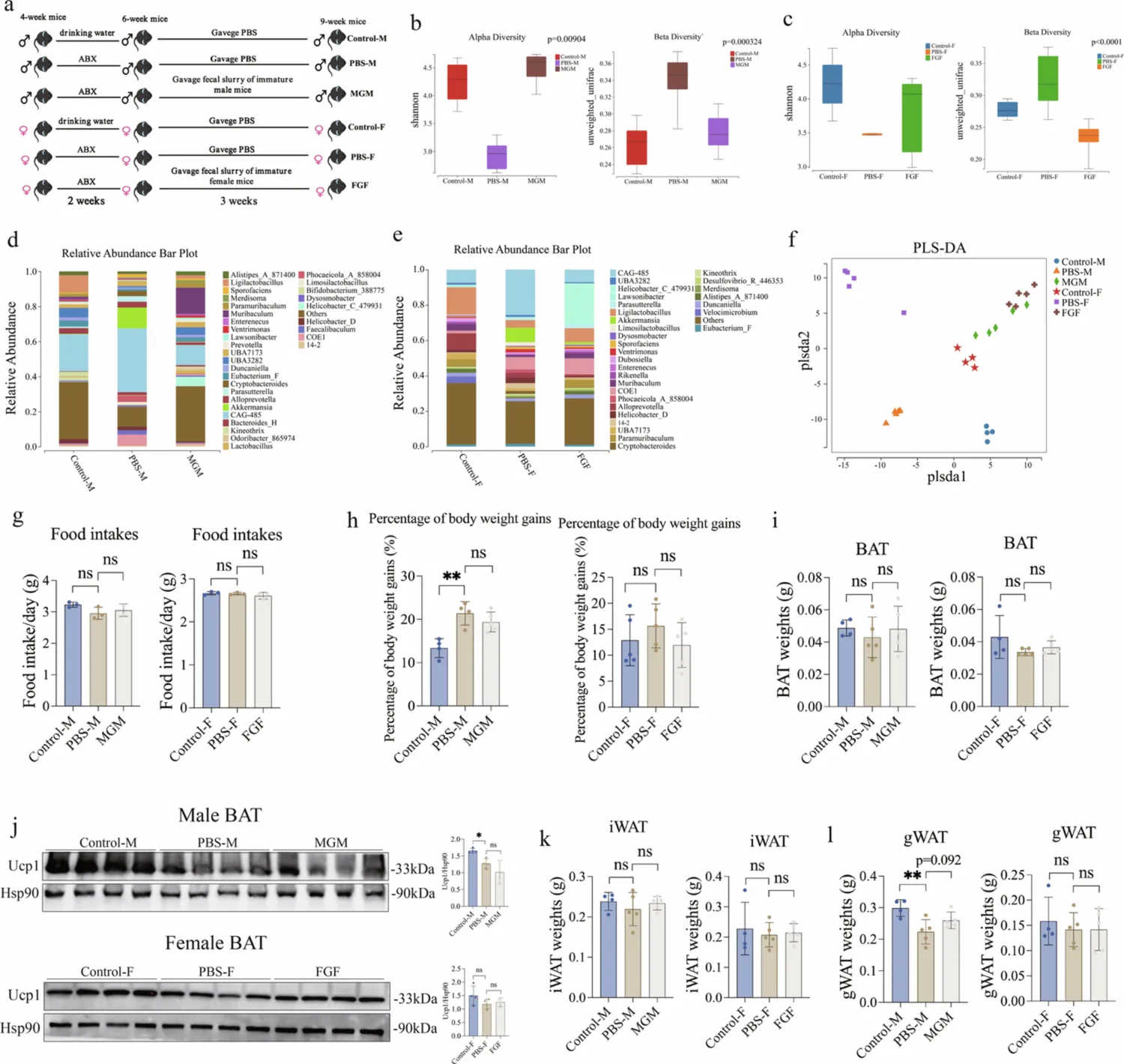
FMT can retrieve ABX-induced gWAT weight loss in male mice. (a) Study design of the FMT experiment. (b) Alpha and beta diversity of fecal-derived gut microbiota among male groups. (c) Alpha and beta diversity of fecal-derived gut microbiota among female groups. (d) Community bar plot analysis of fecal-derived gut microbiota at the phylum level in male mice. (e) Community bar plot analysis of fecal-derived gut microbiota at the phylum level in female mice. (f) PLS-DA plot of fecal-derived gut microbiota. (g) Food intakes, (h) percentage of body weight gains, (i) BAT weights, (j) Ucp1 and Hsp90 protein expression of BAT, (k) iWAT weights, and (l) gWAT weights were compared among different groups in two sexes. For b and c, values are expressed as the median (interquartile range). For g–l, values are expressed as the mean ± sem. *, *p* < 0.05; **, *p* < 0.01; ***, *p* < 0.001; ns, not significant. Abbreviation: ABX, antibiotics; Control-M, ABX-naïve male mice; PBS-M, ABX-treated male mice gavaged with PBS; MGM, ABX-treated male mice gavaged with male mice fecal slurry; Control-F, ABX-naïve female mice; PBS-F, ABX-treated female mice gavaged with PBS; FGF, ABX-treated female mice gavaged with female mice fecal slurry; BAT, brown adipose tissue; iWAT, inguinal white adipose tissue; gWAT, gonadal white adipose tissue.

To determine whether FMT could ameliorate the metabolic alterations induced by short-term ABX exposure, we administered fecal slurry from sex- and age-matched donors to mice following a 2-week ABX regimen (Figure 2a). Microbiota analysis of fecal sample DNA sequencing revealed that FMT restored alpha and beta diversity in ABX-exposed males (Figure 2b), while FMT only restored beta diversity in ABX-exposed females (Figure 2c). But microbiota analysis of cecal content sample DNA sequencing revealed that FMT restored alpha diversity in ABX-exposed males and females (Figure S2d, e, g, h). Taxonomic profiling and PLS-DA of fecal and cecal content microbiota analysis confirmed distinct microbiota restructuring in two sexes following FMT. Additionally, in both male and female mice, FMT failed to completely restore the gut microbiota of ABX‑treated animals to control levels, with significant intergroup differences remaining among the three groups of each sex (Figure 2d–f, Figure S2f, i, j).

Physiologically, neither ABX nor FMT administration affected food intake in either sex (Figure 2g). ABX-exposed male mice receiving PBS (PBS-M) exhibited increased percentage of body weight gains compared to ABX-free male controls (Control-M), an effect not reversed by FMT (Figure 2h). In contrast to male mice in the ABX exposure experiment, there were delayed body weight gains in ABX-exposed and PBS-gavage males (PBS-M) compared to ABX-free and PBS-gavage males (Control-M) in the FMT experiment, which were independent of BAT weights (Figure 2i) but coincided with reduced Ucp1 protein expression in BAT of PBS-M mice vs Control-M mice (Figure 2j). In contrast, females showed no differences in percentage of body weight gains, BAT mass, or UCP1 expression in BAT across groups (Figure 2h–j).

Notably, iWAT mass remained unaltered by ABX and FMT intervention in both sexes (Figure 2k), gWAT mass was significantly reduced by ABX treatment (PBS-M vs Control-M), and this reduction was partially reversed by FMT (MGM vs PBS-M) (Figure 2l). No changes in gWAT mass were observed in female groups (Figure 2l). Serum lipid profiles were unaffected by ABX or FMT in either sex (Figure S3c, d). Furthermore, transcript levels of the fibrosis marker Col1a1 in iWAT depots remained unchanged among groups in both sexes (Figure S3e), while FMT could slightly increase relative Col1a1 mRNA expression in the gWAT of the FGF group when compared to the PBS-F group (Figure S3f). Nonetheless, it suggested that ABX and FMT had no profound impact on adipose tissue fibrogenesis. Hepatic steatosis, fibrosis, and function were largely unaffected by ABX or FMT, except for a reduction in serum ALT levels in FMT-treated females (Figure S3g–k).

### 3.4. Early-life gut dysbiosis exacerbates hepatic steatosis in male HFD-fed mice, a phenotype reversible by FMT

**Figure 3.**
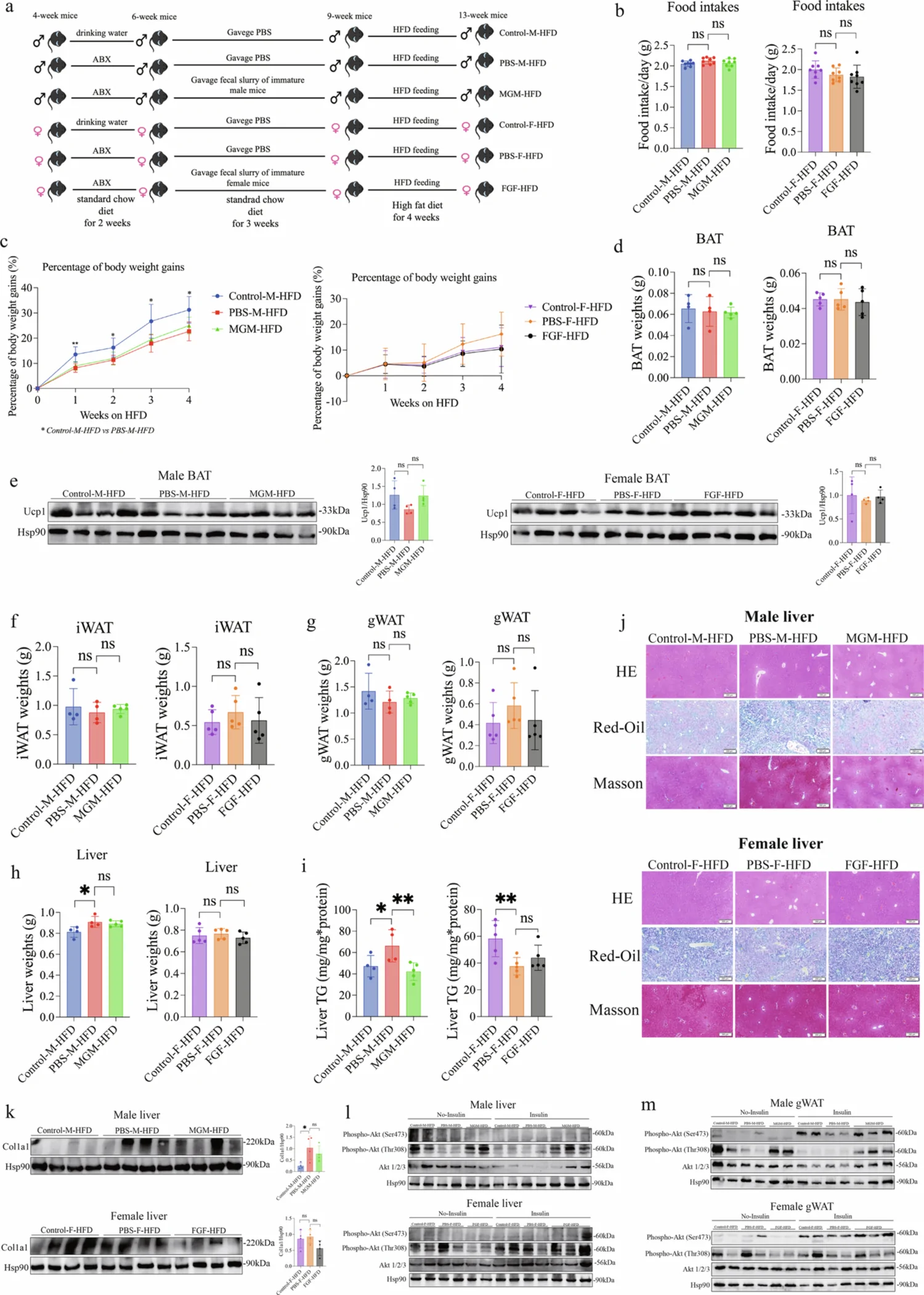
Two-week ABX exposure in early life has sex-dimorphic effects on adiposity, hepatic steatosis, and peripheral insulin sensitivity in HFD mice. (a) Study design of ABX-naïve, ABX-treated, and FMT mice fed with HFD in two sexes. (b) Food intakes, (c) percentage of body weight gains, (d) BAT weights, (e) Ucp1 and Hsp90 protein expression of BAT, (f) iWAT weights, (g) gWAT weights, (h) liver weights, (i) liver TG levels, (j) HE, Red-Oil, Masson staining of liver, (k) Col1a1 and Hsp90 protein expression of liver, (l) Phospho-AKT(Ser473), phosphor-AKT(Thr308), AKT(1/2/3) and Hsp90 protein expression in liver, and (m) gWAT were compared among different groups in two sexes. For b–i and k, values are expressed as the mean ± sem. *, *p* < 0.05; **, *p* < 0.01; ***, *p* < 0.001; ns, not significant. Abbreviation: ABX, antibiotics; TG, triglyceride; Control-M-HFD, ABX-naïve male mice fed with HFD for 4 weeks; PBS-M-HFD, ABX-treated male mice gavaged with PBS fed with HFD for 4 weeks; MGM-HFD, ABX-treated male mice gavaged with male mice fecal slurry fed with HFD for 4 weeks; Control-F-HFD, ABX-naïve female mice fed with HFD for 4 weeks; PBS-F-HFD, ABX-treated female mice gavaged with PBS fed with HFD for 4 weeks; FGF-HFD, ABX-treated female mice gavaged with female mice fecal slurry fed with HFD for 4 weeks; BAT, brown adipose tissue; iWAT, inguinal white adipose tissue; gWAT, gonadal white adipose tissue.

Given the observed reduction in gWAT mass and BAT Ucp1 expression following early-life ABX exposure, which was exhibited only in male mice, we investigated whether these alterations predisposed male and female mice to divergent metabolic dysfunction under HFD challenge (Figure 3a). After 4 weeks of HFD, fecal-derived gut microbiota composition remained distinct among groups in both sexes (Figure S4a–c), but cecal-derived gut microbiota composition became similar in females and remained distinct in males (Figure S2k).

Notably, ABX-exposed and PBS-gavaged HFD male mice (PBS-M-HFD) exhibited significantly lower body weight gain compared to ABX-free and PBS-gavaged HFD male mice (Control-M-HFD), despite comparable food intake (Figure 3b, c). This was not accompanied by changes in BAT mass, morphology, or Ucp1 expression (Figure 3d, e, S4d). In females, no intergroup differences were observed in these parameters (Figure 3b–e, S4d). HFD feeding normalized the previously observed ABX-induced reduction in WAT mass in males, with both iWAT and gWAT weights and morphology showing no differences among groups in both sexes (Figure 3f, g, Figure S4d).

Strikingly, PBS-M-HFD mice displayed increased liver weights and hepatic TG accumulation compared to Control-M-HFD mice, hepatic TG accumulation were attenuated by early-life FMT (Figure 3h, i). While there were no differences in hepatic TG accumulation among male groups in their pre-HFD baseline (Figure S3g). Histological analysis, Red-Oil and Masson staining confirmed exacerbated hepatic steatosis and fibrosis in PBS-M-HFD mice, both of which were ameliorated by FMT (Figure 3j). However, elevated Col1a1 protein expression in PBS-M-HFD livers was not reduced by FMT (Figure 3k). Serum lipid profiles and liver enzyme levels remained largely unchanged in males, though the AST/ALT ratio was marginally elevated in the PBS-M-HFD group compared to Control-M-HFD group (Figure S4e–i).

### 3.5. Early-life ABX exposure attenuates hepatic steatosis in female mice under HFD

In HFD-fed female mice, liver weights remained comparable across groups (Figure 3h). However, ABX-exposed and PBS-gavaged HFD female mice (PBS-F-HFD) showed significantly reduced hepatic TG accumulation compared to ABX-free and PBS-gavaged HFD female mice (Control-F-HFD) (Figure 3i), a contrast to their pre-HFD baseline (Figure S3g). FMT did not reverse or attenuate the ABX‑induced reduction in hepatic triglycerides in females (Figure 3i). This reduction in hepatic lipid deposition was confirmed by HE and Red-Oil staining (Figure 3j). No significant differences were observed in hepatic Col1a1 protein expression (Figure 3k) or serum parameters, including TG, TC, ALT, AST levels, and AST/ALT ratio among female groups (Figure S4e–i).

### 3.6. Early-life gut dysbiosis impaired peripheral insulin sensitivity in young male HFD mice can be reversed by FMT

Given the metabolic alterations in the liver and gWAT, we assessed tissue-specific insulin sensitivity via Akt phosphorylation. In male HFD-fed mice, the PBS-M-HFD group showed markedly reduced phospho-Akt levels (Ser473 and Thr308) in both liver and gWAT following insulin stimulation compared to The Control-M-HFD group, indicating impaired peripheral insulin signaling in male HFD mice with short-term early-life ABX exposure. This defect was substantially rescued by FMT (Figure 3l, m). No significant changes in liver and gWAT phospho-Akt levels (Ser473 and Thr308) were observed in females (Figure 3l, m). Despite these sex-specific alterations, intraperitoneal glucose tolerance tests revealed no differences in systemic glucose homeostasis among groups in either sex (Figure S4j, k), demonstrating that early-life ABX exposure in male mice induces tissue-selective insulin resistance without affecting whole-body glucose tolerance under short-term HFD challenge.

### 3.7. Dimethylarsinous acid (DMAIII) and gut microbiota interactions could mediate ABX-induced adipose remodeling

**Figure 4.**
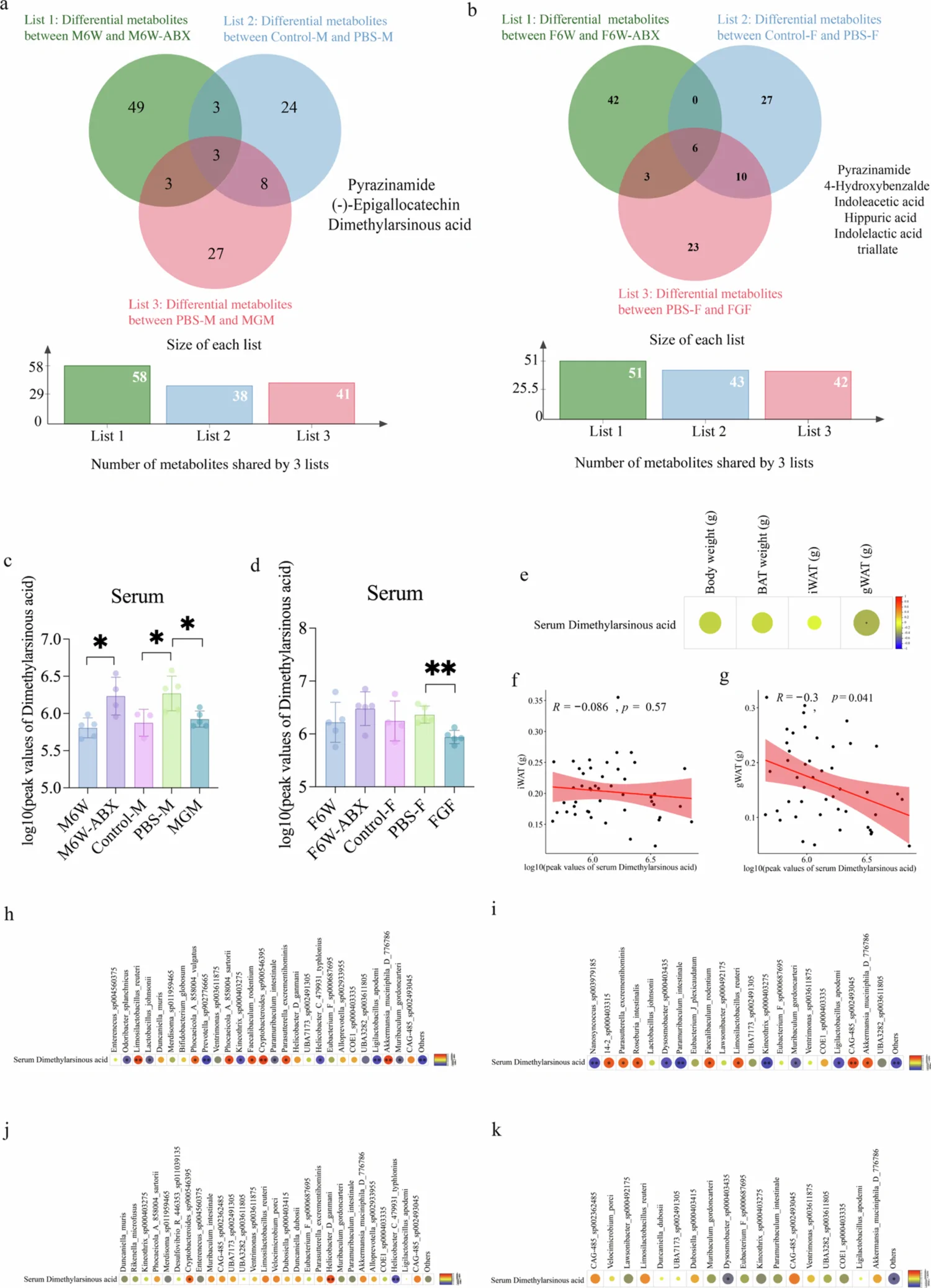
Dimethylarsinous acid is a potential metabolite involved in ABX-induced gWAT weight loss. (a) The intersection of differential metabolites in M6W-ABX vs M6W, PBS-M vs Control-M, and MGM vs PBS-M. (b) The intersection of differential metabolites in F6W-ABX vs F6W, PBS-F vs Control-F, and FGF vs PBS-F. (c) Log10 of peak values of serum dimethylarsinous acid among different groups in males. (d) Log10 of peak values of serum dimethylarsinous acid among different groups in females. (e) Correlation analyses between Log10 of peak values of serum dimethylarsinous acid and adiposity indices. (f) Scatter plot of Log10 of peak values of serum dimethylarsinous acid and iWAT weights. (g) Scatter plot of Log10 of peak values of serum dimethylarsinous acid and gWAT weights. Correlation analyses between Log10 of peak values of serum dimethylarsinous acid and the relative abundance of fecal-derived (h) and cecal-derived (i) gut bacterial species in male mice from FMT experiments. Correlation analyses between Log10 of peak values of serum dimethylarsinous acid and the relative abundance of fecal-derived (j) and cecal-derived (k) gut bacterial species in female mice from FMT experiments. For c and d, values are expressed as the mean ± sem. *, *p* < 0.05; **, *p* < 0.01; ***, *p* < 0.001; ns, not significant. Abbreviation: M6W, ABX-naïve male mice; F6W, ABX-naïve female mice; M6W-ABX, ABX-treated male mice; F6W-ABX, ABX-treated female mice; Control-M, ABX-naïve male mice; PBS-M, ABX-treated male mice gavaged with PBS; MGM, ABX-treated male mice gavaged with male mice fecal slurry; Control-F, ABX-naïve female mice; PBS-F, ABX-treated female mice gavaged with PBS; FGF, ABX-treated female mice gavaged with female mice fecal slurry; BAT, brown adipose tissue; iWAT, inguinal white adipose tissue; gWAT, gonadal white adipose tissue.

To identify mediators linking gut microbiota alterations to metabolic phenotypes, we integrated serum metabolomic and microbial profiling data from ABX- and FMT-treated mice. Differential analysis revealed sex-specific changes in serum metabolites (Figure S5a–f), fecal-derived bacterial species (Figure S5g–j), and cecal-derived bacterial species (Figure S6a–d). Firstly, we focused on serum metabolites exhibiting consistent reversal patterns in males: significantly altered by ABX and normalized by FMT, while remaining stable in females. This screening identified only DMAIII as a candidate mediator (Figure 4a, b). Serum DMAIII levels were elevated following ABX exposure and reduced by FMT in males (Figure 4c), while remaining unchanged by ABX exposure but decreased by FMT in females (Figure 4d). It was worth noting that serum DMAIII levels showed an inverse correlation with gWAT mass; no such correlations were observed with body weight or other adipose depots (Figure 4e–g).

Among the highly abundant fecal-derived gut bacteria in the FMT male model, *CAG-485 sp002493045*, *Muribaculum gordoncarteri, Akkermansia muciniphila D_776786,* *Ligilactobacillus apodemi* and *UBA3282 sp003611805* were perturbed by ABX and subsequently restored by FMT (Figure S5h), among which, only *CAG-485 sp002493045*, *Muribaculum gordoncarteri,* and *Ligilactobacillus apodemi* were highly abundant in the ABX-treated mice model (Figure S5g). In the ABX and FMT models, the relative abundance of fecal-derived *Muribaculum gordoncarteri* in male mice was consistently and effectively suppressed by ABX and simultaneously restored by FMT (Figure S5g, h). Although no change in the relative abundance of *Ligilactobacillus apodemi* was observed immediately after 2 weeks of ABX treatment, it was reduced by ABX and increased by FMT when assessed 3 weeks after the cessation of ABX treatment in the FMT male model (Figure S5g, h). The relative abundance of *CAG-485 sp002493045* was suppressed by ABX in the ABX model, whereas in the FMT model, ABX treatment paradoxically increased its abundance (Figure S5g, h). Furthermore, among these species mentioned above, serum DMAIII levels were correlated inversely with the abundance of *Muribaculum gordoncarteri* and *Ligilactobacillus apodemi* and were correlated positively with the abundance of *Akkermansia muciniphila D_776786* in feces (Figure 4h).

Additionally, similar changes were observed in the cecal-derived gut microbiota of male mice (Figure S6a, b). *UBA3282 sp003611805*, *Akkermansia muciniphila D_776786*, *Ligilactobacillus apodemi*, *CAG-485 sp002493045*, *Ventrimonas sp003611875*, *Muribaculum gordoncarteri,* and *Kineothrix sp000403275* were significantly altered by ABX and restored by FMT (Figure S6b), among which, only *UBA3282 sp003611805*, *Akkermansia muciniphila D_776786*, *Ligilactobacillus apodemi*, *Muribaculum gordoncarteri,* and *Kineothrix sp000403275* were highly abundant in the cecal content of ABX-treated male mice model (Figure S6a). Notably, *UBA3282 sp003611805*, *Muribaculum gordoncarteri*, and *Kineothrix sp000403275* were consistently suppressed by ABX and simultaneously restored by FMT in these two models (Figure S6a, b), while the relative abundance of *Akkermansia muciniphila D_776786* and *Ligilactobacillus apodemi* were not changed by 2-week ABX intervention in the ABX model (Figure S6a). Among these bacterial species mentioned above, serum DMAIII levels were correlated inversely with the relative abundances of *Ligilactobacillus apodemi*, *Muribaculum gordoncarteri*, and *Kineothrix sp000403275* and were correlated positively with abundances of *Akkermansia muciniphila D_776786* and *CAG-485 sp002493045* in cecal content (Figure 4i).

Applying the same analytical strategy to the female mice model, we identified several fecal bacteria, including *CAG-485 sp002493045*, *Akkermansia muciniphila D_76786*, *Paramuribaculum intestinale*, *Muribaculum gordoncarteri,* and *Helicobacter D ganmani,* whose responses to ABX and FMT were opposite in direction (Figure S5j). Among these bacterial species, the relative abundances of *Paramuribaculum intestinale* and *Muribaculum gordoncarteri* exhibited suppressed effects by ABX treatment in the ABX exposure experiment (Figure S5i). However, none of these fecal-derived bacteria mentioned above showed a significant correlation with serum DMAIII levels (Figure 4j). In the cecal-derived gut microbiota of female mice, *Akkermansia muciniphila D_76786*, *Ligilactobacillus apodemi*, *Ventrimonas sp003611875*, *Paramuribaculum intestinale*, *Kineothrix sp000403275*, and *Dysosmobacter sp000403435* displayed opposing responses to ABX and FMT treatment in the FMT model (Figure S6d). Of these, *Kineothrix sp000403275* was consistently suppressed in the ABX model (Figure S6c). Notably, among cecal-derived bacteria mentioned above, only *Dysosmobacter sp000403435* exhibited a negative correlation with serum DMAIII levels in females (Figure 4k).

Compared to male mice, the interaction between the gut microbiota and serum DMAIII was considerably weaker in female mice, which may explain why ABX treatment did not significantly affect serum DMAIII levels in females. Collectively, our findings indicate that multiple bacteria in the gut of both male and female mice may be involved in DMAIII metabolism. Integrated analyses of fecal- and cecal-derived gut microbiota combined with metabolomics data suggest that *Muribaculum gordoncarteri* and *Ligilactobacillus apodemi* may play an important role in the catabolic regulation of DMAIII, which warrants further experimental investigation in future studies.

### 3.8. Sex-specific metabolite profiles associated with hepatic steatosis in HFD-fed mice

**Figure 5.**
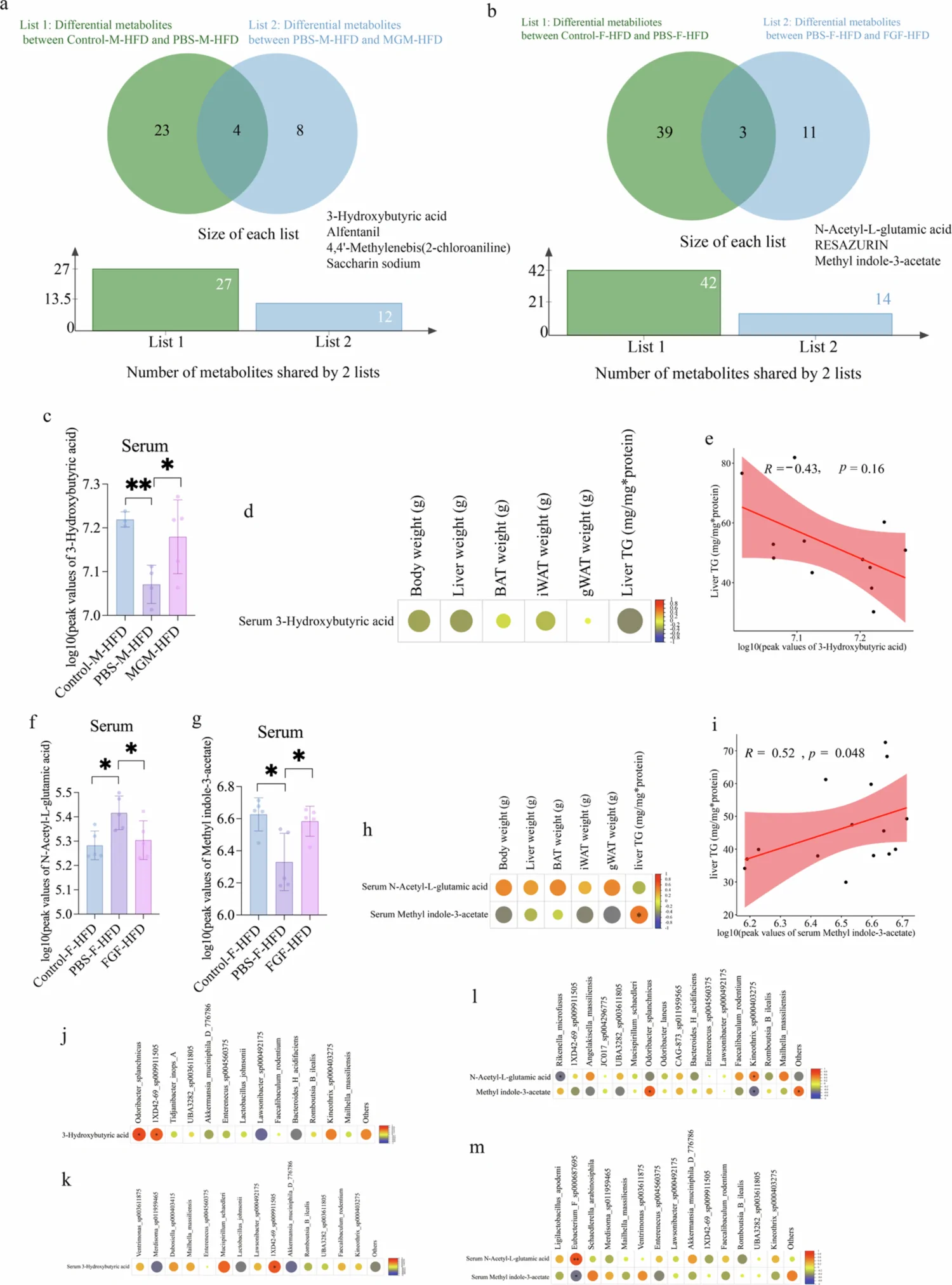
Potential metabolites involved in ABX- and FMT-induced liver TG changes in male and female mice fed with HFD for 4 weeks. (a) The intersection of differential metabolites in PBS-M-HFD vs Control-M-HFD and MGM-HFD vs PBS-M-HFD. (b) The intersection of differential metabolites in PBS-F-HFD vs Control-F-HFD and FGF-HFD vs PBS-F-HFD. (c) Log10 of peak values of serum 3-Hydroxybutyric acid among different groups in male HFD mice. (d) Correlation analyses of Log10 of peak values of serum 3-Hydroxybutyric acid with physiological indices and liver TG in male HFD mice. (e) Scatter plot of Log10 of peak values of serum 3-Hydroxybutyric acid and liver TG levels in male HFD mice. (f) Log10 of peak values of *N*-Acetyl-L-glutamic acid among different groups in female HFD mice. (g) Log10 of peak values of Methyl indole-3-acetate among different groups in female HFD mice. (h) Correlation analyses of Log10 of peak values of serum *N*-Acetyl-L-glutamic acid and Methyl indole-3-acetate with physiological indices and liver TG in female HFD mice. (i) Scatter plot of Log10 of peak values of serum Methyl indole-3-acetate and liver TG levels in female HFD mice. The correlation analysis between Log10 of peak values of serum 3-Hydroxybutyric acid and fecal-derived (j) and cecal-derived (k) gut bacterial species in male HFD mice. The correlation analysis of Log10 of peak values of serum *N*-Acetyl-L-glutamic acid and Methyl indole-3-acetate with fecal-derived (l) and cecal-derived (m) gut bacterial species in female HFD mice. For c, f, and g, values are expressed as the mean ± sem. *, *p* < 0.05; **, *p* < 0.01; ***, *p* < 0.001; ns, not significant. Abbreviation: TG, triglyceride; Control-M-HFD, ABX-naïve male mice fed with HFD for 4 weeks; PBS-M-HFD, ABX-treated male mice gavaged with PBS fed with HFD for 4 weeks; MGM-HFD, ABX-treated male mice gavaged with male mice fecal slurry fed with HFD for 4 weeks; Control-F-HFD, ABX-naïve female mice fed with HFD for 4 weeks; PBS-F-HFD, ABX-treated female mice gavaged with PBS fed with HFD for 4 weeks; FGF-HFD, ABX-treated female mice gavaged with female mice fecal slurry fed with HFD for 4 weeks; BAT, brown adipose tissue; iWAT, inguinal white adipose tissue; gWAT, gonadal white adipose tissue.

To elucidate the metabolic basis of sexually dimorphic phenotypes, we performed serum untargeted metabolomics in HFD-fed mice. In males, four metabolites were consistently altered by both early-life ABX exposure and subsequent FMT (Figure 5a). Alfentanil was a potent, rapid-onset, and short-duration synthetic opioid analgesic. 4,4'-Methylenebis(2-chloroaniline) is a synthetic organic compound and an industrial intermediate. It is a confirmed human carcinogen and primarily causes bladder cancer. Saccharin sodium was the sodium salt of saccharin, one of the earliest discovered artificial sweeteners. It essentially could not be metabolized *in vivo*, and the majority of it was excreted unchanged in the urine within 24 h after absorption. 3-Hydroxybutyric acid was an organic acid and the most abundant of the three "ketone bodies." It was produced in the liver from the oxidation of fatty acids, particularly under conditions of inadequate carbohydrate intake or when carbohydrates could not be utilized effectively. After excluding xenobiotics (alfentanil, 4,4'-methylenebis(2-chloroaniline), and saccharin sodium), 3-hydroxybutyric acid emerged as a candidate mediator. Although its serum levels decreased with ABX and increased after FMT (Figure 5c), no significant correlation was found with hepatic triglyceride (TG) accumulation or other metabolic parameters (Figure 5d, e).

In females, three metabolites met the same screening criteria (Figure 5b). Resazurin was excluded as a technical artifact, while *N*-Acetyl-L-glutamic acid and methyl indole-3-acetate showed reciprocal patterns: ABX increased *N*-Acetyl-L-glutamic acid and decreased methyl indole-3-acetate levels, with FMT reversing these alterations (Figure 5f, g). Notably, only serum methyl indole-3-acetate demonstrated a significant positive correlation with hepatic TG content (Figure 5h, i).

Microbial correlation analysis revealed that serum 3-hydroxybutyric acid levels were positively correlated with the relative abundance of fecal and cecal-derived *1XD42-69 sp009911505* in male HFD mice (Figure 5j, k). However, the relative abundance of *1XD42-69 sp009911505* was not in the top 10 high in fecal microbiota (Figure S4b) or significantly changed by ABX and FMT in cecal microbiota (Figure S6e). *Bacteroides H acidifaciens* in the feces of HFD male mice were affected by ABX and FMT in an opposite direction (Figure S4b). In the cecal content of HFD male mice, *Akkermansia muciniphila D_776786*, *Lactobacillus johnsonii*, and *Mucispirillum schaedleri* were affected by ABX and FMT in an opposite direction (Figure S6e).

In female mice, serum *N*-Acetyl-L-glutamic acid levels were negatively associated with the relative abundance of *Rikenella microfusus* and were positively associated with the relative abundance of *Kineothrix sp000403275* in feces (Figure 5l), but were only positively associated with *Eubacterium F sp000687695* in cecal content (Figure 5m). However, serum methyl indole-3-acetate levels were positively correlated to *Odoribacter splanchnicus* and were negatively correlated to *Kineothrix sp000403275* in fecal-derived microbiota (Figure 5l), which also had a significant but negative correlation with *Eubacterium F sp000687695* in cecal microbiota (Figure 5m). These indicate that *Kineothrix sp000403275* and *Eubacterium F sp000687695* may be involved in the metabolism of both *N*-Acetyl-L-glutamic acid and methyl indole-3-acetate (Figure 5l, m). Correspondingly, the relative abundance of *Kineothrix sp000403275* was increased by ABX but was decreased by FMT in the feces of female HFD mice (Figure S4c), and the relative abundance of *1XD42-69 sp009911505* was changed oppositely by ABX and FMT in the cecal content of HFD female mice (Figure S6f).

In summary, there appeared to be no pronounced interaction between 3-hydroxybutyric acid and the gut microbiota in male HFD mice. In female HFD mice, *Kineothrix sp000403275,* an uncultured bacterium, may attenuate hepatic lipid accumulation via its involvement in the metabolism of *N*-Acetyl-L-glutamic acid and methyl indole-3-acetate. Targeted experimental approaches, such as cultivation-based studies or gnotobiotic models, are needed to validate the proposed metabolic interactions

## 4. Discussion

Unlike previous investigations emphasizing prolonged antibiotic regimens, the present study reveals that even transient ABX exposure induces sexually dimorphic metabolic alterations. Specifically, a 2-week ABX course suppressed gWAT accumulation in immature male mice, an effect associated with exacerbated hepatic steatosis and fibrosis in later life under dietary challenge. These adverse outcomes were partially reversible via FMT administered post-ABX. Male mice further exhibited delayed suppression of brown adipose tissue activity after cessation of ABX treatment for 3 weeks, reflected by altered body weight trajectories, whereas females remained metabolically unaffected, underscoring a pronounced sex-specific response to short-term microbiome perturbation.

Sex differences in gut microbiota composition have been established in sexually mature hosts,^9^ yet emerging evidence suggests their developmental origins precede puberty. Human studies indicate diminished microbial sex differences in elderly populations, implying hormonal involvement.^10^ Recent work demonstrates that microbiota divergence initiates before pubertal onset and intensifies during maturation.^11^ ^,^ ^12^ Longitudinal analyses reveal that female gut microbiota progressively adopt adult-like characteristics during puberty.^13^ It has also been found in murine models where post-pubertal animals exhibit distinct sex-specific microbial profiles.^14^ Crucially, gonadectomy experiments provide direct evidence for sex hormones mediating these compositional differences, highlighting the dynamic interplay between host development and microbial community assembly.^15^

Our longitudinal analysis of C57BL/6 mice revealed sexually dimorphic gut microbiota configurations across developmental stages, from weaning (3 weeks) through young adulthood (8 weeks)-even during periods of low sex hormone influence. These findings underscore the necessity of accounting for sex as a biological variable in gut dysbiosis research, including studies focused on early-life microbial perturbations in immature animal models.

It has been demonstrated that prolonged antibiotic regimens (>4 weeks) consistently promoted body weight gain and metabolic dysfunction in murine models,^7^ ^,^ ^8^ ^,^ ^16^ such that extended exposures poorly reflect typical clinical courses, particularly in pediatric populations. Our adoption of a physiologically relevant 2-week ABX regimen revealed sexually dimorphic metabolic responses distinct from those associated with chronic exposure, underscoring the temporal and sex-specific nature of microbiota-mediated metabolic regulation.

Our untargeted metabolomics identified serum dimethylarsinous acid (DMAIII) as a key mediator of ABX-induced suppression of WAT accumulation. Humans and animals are exposed to inorganic arsenic primarily via dietary intake, including the consumption of contaminated water and food.^17-20^ In humans and some animals, iAs is metabolized in a series of methylation steps catalyzed by arsenic (+3 oxidation state) methyltransferase (AS3MT), forming monomethylarsenic (MAs) and dimethylarsenic (DMAs), which are excreted primarily in the urine. The metabolism of iAs is generally thought to occur primarily in the liver.^21^ ^,^ ^22^ DMAIII is a highly reactive trivalent intermediate in inorganic arsenic metabolism, exerting toxicity through strong nucleophilic binding to protein thiol groups.^23^ This interaction potently inhibits key metabolic enzymes, including pyruvate dehydrogenase and *α*-ketoglutarate dehydrogenase, disrupting cellular energy pathways and potentially explaining the observed impairment in lipid storage. Supporting this mechanism, As3mt-knockout mice exposed to inorganic arsenic display increased adiposity, consistent with disrupted arsenic metabolism promoting fat accumulation.^24^

The inverse correlation between serum DMAIII levels and the abundance of *Muribaculum gordoncarteri* and *Ligilactobacillus apodemi* suggests these taxa may participate in DMAIII degradation. FMT reduced DMAIII in females, but minimal gWAT deposition occurs during the post-pubertal stage, potentially explaining the absence of measurable effects on gWAT mass in female mice.

Human adipocyte expansion occurs predominantly during infancy and adolescence. Compromised early adipose development may trigger adipocyte hypertrophy in later life, exacerbating insulin resistance.^25^ This concept is supported by the Finnish Young Finns Study, which linked reduced childhood visceral fat with compensatory subcutaneous expansion to impaired metabolic flexibility in adulthood.^26^ Similarly, prenatal or childhood famine exposure increases susceptibility to adult obesity, insulin resistance, and cardiovascular disease, collectively illustrating how early metabolic programming under nutritional constraint predisposes to dysregulated fat storage upon subsequent nutrient abundance.^27^

The observed reduction in gWAT mass following early-life ABX exposure likely contributes to the subsequent exacerbation of hepatic steatosis under HFD conditions, a phenomenon consistent with the established role of impaired visceral adipose expandability in fatty liver pathogenesis. This concept is further supported by the partial reversal of both gWAT accumulation and hepatic lipid deposition through FMT. The concurrent reduction in body weight gain in PBS-M-HFD mice, despite unchanged food intake and BAT activity, may reflect ABX-induced impairments in energy harvest^28^ ^,^ ^29^ and gut barrier integrity.^30^ ^,^ ^31^ While serum 3-hydroxybutyrate levels were modulated by both ABX and FMT, their lack of correlation with other metabolic parameters suggests they may represent a secondary consequence of altered lipid handling rather than a primary driver of hepatic steatosis.

In female mice, baseline WAT mass and hepatic TG levels remained comparable across groups before HFD challenge, as did food intake, BAT activity, and body weight gain following HFD feeding. Notably, PBS-F-HFD mice displayed reduced hepatic TG accumulation, a phenomenon potentially linked to decreased serum methyl indole-3-acetate, a stable auxin precursor. The correlation between serum methyl indole-3-acetate levels and the relative abundance of *Kineothrix sp000403275*, an uncultured member of the genus *Kineothrix*, further suggests this taxon may participate in its microbial metabolism and exert beneficial effects on hepatic steatosis. This potential regulatory axis warrants further investigation.

In summary, this work delineates a previously uncharacterized metabolic consequence of short-term early-life ABX exposure: suppression of visceral WAT expansion in immature male mice, which predisposes to exacerbated hepatic steatosis and fibrosis under dietary challenge, a phenotype reversible through timely microbiota restoration. Conversely, female mice exhibited protected hepatic lipid metabolism following the same intervention. Integrated multi-omics revealed candidate metabolites and bacterial taxa potentially governing these sexually dimorphic responses, providing a framework for future mechanistic investigation. Collectively, our findings underscore the necessity of incorporating sex, intervention window, and duration of ABX as fundamental variables in microbiota-metabolic research.

## Supplementary Material

Supplementary Material Supplementary Material

supplementary figures.xlsx supplementary figures.xlsx

main figures.xlsx main figures.xlsx

## Data Availability

The authors declare that all data supporting the findings of this study are available within the paper and its supplementary information files. The raw sequencing data are available in the NCBI Sequence Read Archive (SRA) repository under the BioProject accession number PRJNA1479465, and the raw metabolomics data are available in the MetaboLights repository under the accession number MTBLS14801.

## References

[1] Yatsunenko T , Rey FE , Manary MJ , Trehan I , Dominguez-Bello MG , Contreras M , Magris M , Hidalgo G , Baldassano RN , Anokhin AP , et al. Human gut microbiome viewed across age and geography. Nature. 2012;486(7402):222–227. doi: 10.1038/nature11053. 22699611 PMC3376388

[2] Bezirtzoglou E . The intestinal microflora during the first weeks of life. Anaerobe. 1997;3(2-3):173–177. doi: 10.1006/anae.1997.0102. 16887585

[3] Vangay P , Ward T , Gerber JS , Knights D . Antibiotics, pediatric dysbiosis, and disease. Cell Host Microbe. 2015;17(5):553–564. doi: 10.1016/j.chom.2015.04.006. 25974298 PMC5555213

[4] Parkin K , Christophersen CT , Verhasselt V , Cooper MN , Martino D . Risk factors for gut dysbiosis in early life. Microorganisms. 2021;9(10):2066. doi: 10.3390/microorganisms9102066. 34683389 PMC8541535

[5] Trasande L , Blustein J , Liu M , Corwin E , Cox LM , Blaser MJ . Infant antibiotic exposures and early-life body mass. Int J Obes (Lond). 2013;37(1):16–23. doi: 10.1038/ijo.2012.132. 22907693 PMC3798029

[6] Murphy R , Stewart AW , Braithwaite I , Beasley R , Hancox RJ , Mitchell EA . Antibiotic treatment during infancy and increased body mass index in boys: an international cross-sectional study. Int J Obes (Lond). 2014;38(8):1115–1119. doi: 10.1038/ijo.2013.218. 24257411

[7] Cho I , Yamanishi S , Cox L , Methé BA , Zavadil J , Li K , Gao Z , Mahana D , Raju K , Teitler I , et al. Antibiotics in early life alter the murine colonic microbiome and adiposity. Nature. 2012;488(7413):621–626. doi: 10.1038/nature11400. 22914093 PMC3553221

[8] Cox LM , Yamanishi S , Sohn J , Alekseyenko AV , Leung JM , Cho I , Kim SG , Li H , Gao Z , Mahana D , et al. Altering the intestinal microbiota during a critical developmental window has lasting metabolic consequences. Cell. 2014;158(4):705–721. doi: 10.1016/j.cell.2014.05.052. 25126780 PMC4134513

[9] Santos-Marcos JA , Mora-Ortiz M , Tena-Sempere M , Lopez-Miranda J , Camargo A . Interaction between gut microbiota and sex hormones and their relation to sexual dimorphism in metabolic diseases. Biol Sex Differ. 2023;14(1):4. doi: 10.1186/s13293-023-00490-2. 36750874 PMC9903633

[10] Zhang X , Zhong H , Li Y , Shi Z , Ren H , Zhang Z , Zhou X , Tang S , Han X , Lin Y , et al. Sex- and age-related trajectories of the adult human gut microbiota shared across populations of different ethnicities. Nat Aging. 2021;1(1):87–100. doi: 10.1038/s43587-020-00014-2. 37118004

[11] Yuan X , Chen R , Zhang Y , Lin X , Yang X . Gut microbiota: effect of pubertal status. BMC Microbiol. 2020;20(1):334. doi: 10.1186/s12866-020-02021-0. 33143658 PMC7640488

[12] Yuan X , Chen R , Zhang Y , Lin X , Yang X . Sexual dimorphism of gut microbiota at different pubertal status. Microb Cell Fact. 2020;19(1):152. doi: 10.1186/s12934-020-01412-2. 32723385 PMC7390191

[13] Korpela K , Kallio S , Salonen A , Hero M , Kukkonen AK , Miettinen PJ , Savilahti E , Kohva E , Kariola L , Suutela M , et al. Gut microbiota develop towards an adult profile in a sex-specific manner during puberty. Sci Rep. 2021;11(1):23297. doi: 10.1038/s41598-021-02375-z. 34857814 PMC8640005

[14] Yurkovetskiy L , Burrows M , Khan AA , Graham L , Volchkov P , Becker L , Antonopoulos D , Umesaki Y , Chervonsky AV . Gender bias in autoimmunity is influenced by microbiota. Immunity. 2013;39(2):400–412. doi: 10.1016/j.immuni.2013.08.013. 23973225 PMC3822899

[15] Org E , Mehrabian M , Parks BW , Shipkova P , Liu X , Drake TA , Lusis AJ . Sex differences and hormonal effects on gut microbiota composition in mice. Gut Microbes. 2016;7(4):313–322. doi: 10.1080/19490976.2016.1203502. 27355107 PMC4988450

[16] Mahana D , Trent CM , Kurtz ZD , Bokulich NA , Battaglia T , Chung J , Müller CL , Li H , Bonneau RA , Blaser MJ . Antibiotic perturbation of the murine gut microbiome enhances the adiposity, insulin resistance, and liver disease associated with high-fat diet. Genome Med. 2016;8(1):48. doi: 10.1186/s13073-016-0297-9. 27124954 PMC4847194

[17] Lynch HN , Greenberg GI , Pollock MC , Lewis AS . A comprehensive evaluation of inorganic arsenic in food and considerations for dietary intake analyses. Sci Total Environ. 2014;496:299–313. doi: 10.1016/j.scitotenv.2014.07.032. 25089691

[18] Steinmaus C , Carrigan K , Kalman D , Atallah R , Yuan Y , Smith AH . Dietary intake and arsenic methylation in a U.S. Population. Environ Health Perspect. 2005;113(9):1153–1159. doi: 10.1289/ehp.7907. 16140620 PMC1280394

[19] Gu Z , de Silva S , Reichman SM . Arsenic concentrations and dietary exposure in rice-based infant food in Australia. Int J Environ Res Public Health. 2020;17(2):415. doi: 10.3390/ijerph17020415. 31936289 PMC7014030

[20] Yoshinaga J , Narukawa T . Association of dietary intake and urinary excretion of inorganic arsenic in the Japanese subjects. Regul Toxicol Pharmacol. 2020;116:104745. doi: 10.1016/j.yrtph.2020.104745. 32712302

[21] Thomas DJ , Nava GM , Cai S-Y , Boyer JL , Hernández-Zavala A , Gaskins HR . Arsenic (+3 Oxidation State) methyltransferase and the methylation of arsenicals in the invertebrate chordate ciona intestinalis. Toxicol Sci. 2009;113(1):70–76. doi: 10.1093/toxsci/kfp250. 19833739 PMC2902911

[22] Thomas DJ , Styblo M , Lin S . The cellular metabolism and systemic toxicity of arsenic. Toxicol Appl Pharmacol. 2001;176(2):127–144. doi: 10.1006/taap.2001.9258. 11601889

[23] Léonard A , Lauwerys RR . Carcinogenicity, teratogenicity and mutagenicity of arsenic. Mutat Res. 1980;75(1):49–62. doi: 10.1016/0165-1110(80)90027-5. 6987506

[24] Huang MC , Douillet C , Dover EN , Zhang C , Beck R , Tejan-Sie A , Krupenko SA , Stýblo M . Metabolic phenotype of wild-type and As3mt-Knockout C57BL/6J mice exposed to inorganic arsenic: the role of dietary fat and folate intake. Environ Health Perspect. 2018;126(12):127003. doi: 10.1289/ehp3951. 30675811 PMC6371649

[25] Spalding KL , Arner E , Westermark PO , Bernard S , Buchholz BA , Bergmann O , Blomqvist L , Hoffstedt J , Näslund E , Britton T , et al. Dynamics of fat cell turnover in humans. Nature. 2008;453(7196):783–787. doi: 10.1038/nature06902. 18454136

[26] Pulkki-Råback L , Elovainio M , Hakulinen C , Lipsanen J , Hintsanen M , Jokela M , Kubzansky LD , Hintsa T , Serlachius A , Laitinen TT , et al. Cumulative effect of psychosocial factors in youth on ideal cardiovascular health in adulthood: the cardiovascular risk in young Finns study. Circulation. 2015;131(3):245–253. doi: 10.1161/circulationaha.113.007104. 25583139

[27] Ravelli GP , Stein ZA , Susser MW . Obesity in young men after famine exposure in utero and early infancy. NEJM. 1976;295(7):349–353. doi: 10.1056/nejm197608122950701. 934222

[28] Angelakis E , Merhej V , Raoult D . Related actions of probiotics and antibiotics on gut microbiota and weight modification. Lancet Infect Dis. 2013;13(10):889–899. doi: 10.1016/s1473-3099(13)70179-8. 24070562

[29] John GK , Mullin GE . The gut microbiome and obesity. Curr Oncol Rep. 2016;18(7):45. doi: 10.1007/s11912-016-0528-7. 27255389

[30] Malesza IJ , Malesza M , Walkowiak J , Mussin N , Walkowiak D , Aringazina R , Bartkowiak-Wieczorek J , Mądry E . High-fat, Western-style diet, systemic inflammation, and gut microbiota: a narrative review. Cells. 2021;10(11):3164. doi: 10.3390/cells10113164. 34831387 PMC8619527

[31] Rohr MW , Narasimhulu CA , Rudeski-Rohr TA , Parthasarathy S . Negative effects of a high-fat diet on intestinal permeability: a review. Adv Nutr. 2020;11(1):77–91. doi: 10.1093/advances/nmz061. 31268137 PMC7442371

