# Supplementary material for: Early-life gut dysbiosis programs divergent metabolic trajectories in a sex-dependent manner
Source: Gut Microbes. 2026 Aug 20;18(1):2718681. doi: 10.1080/19490976.2026.2718681 (PMC13502010; doi:10.1080/19490976.2026.2718681)
Supplement: Supplementary file 1 [file KGMI_A_2718681_SM8208.docx]

**Supplementary Figure 1.** **Immature mice have sex-dimorphic gut microbiota.** (a) Species accumulation curve of 16S sequencing. (b) Rank-abundance curve of OTUs. (c-j) Alpha and beta diversity of fecal-derived gut microbiota in male and female C57BL/6 mice aged 3-8 weeks. (k) Community bar plot analysis of fecal-derived gut microbiota at the phylum level. (l) Community bar plot analysis of fecal-derived gut microbiota at the genus level. (m-p) PLS-DA plot of fecal-derived gut microbiota in male and female C57BL/6 mice aged 3-8 weeks. For c-j, values are expressed as the median (interquartile range). *, p<0.05; **, p<0.01; ***, p<0.001; ns, not significant. Abbreviation: M3W, male mice aged three weeks; F3W, female mice aged three weeks; M4W, male mice aged four weeks; F4W, female mice aged four weeks; M6W, male mice aged six weeks; F6W, female mice aged six weeks; M8W, male mice aged eight weeks; F8W, female mice aged eight weeks.

**Supplementary Figure 2.** **Cecal-derived gut microbiota composition in the ABX exposure experiment, FMT experiment and HFD experiment.** (a) Alpha diversity and (b) beta diversity of cecal-derived gut microbiota in C57BL/6 mice between ABX-naïve and ABX-treated mice in two sexes. (c) PLS-DA plot of cecal-derived gut microbiota in ABX exposure experiment. (d-e) Alpha and beta diversity diversity of cecal-derived gut microbiota in C57BL/6 male mice in FMT experiment. (g) Community bar plot analysis of cecal-derived gut microbiota at the genus level in males. (g-h) Alpha and beta diversity of cecal-derived gut microbiota in C57BL/6 female mice in FMT experiment. (i) Community bar plot analysis of cecal-derived gut microbiota at the genus level in females. (j) PLS-DA plot of cecal-derived gut microbiota in the FMT experiment. (k) PLS-DA plot of cecal-derived gut microbiota in the HFD experiment. Values are expressed as the median (interquartile range). *, p<0.05; **, p<0.01; ***, p<0.001; ns, not significant. Abbreviation: M6W, ABX-naïve male mice; F6W, ABX-naïve female mice; M6W-ABX, ABX-treated male mice; F6W-ABX, ABX-treated female mice; Control-M, ABX-naïve male mice; PBS-M, ABX-treated male mice gavaged with PBS; MGM, ABX-treated male mice gavaged with male mice fecal slurry; Control-F, ABX-naïve female mice; PBS-F, ABX-treated female mice gavaged with PBS; FGF, ABX-treated female mice gavaged with female mice fecal slurry; Control-M-HFD, ABX-naïve male mice fed with HFD for four weeks; PBS-M-HFD, ABX-treated male mice gavaged with PBS fed with HFD for four weeks; MGM-HFD, ABX-treated male mice gavaged with male mice fecal slurry fed with HFD for four weeks; Control-F-HFD, ABX-naïve female mice fed with HFD for four weeks; PBS-F-HFD, ABX-treated female mice gavaged with PBS fed with HFD for four weeks; FGF-HFD, ABX-treated female mice gavaged with female mice fecal slurry fed with HFD for four weeks.

**Supplementary Figure 3.** **Physiological and biochemical indicators of mice in the ABX exposure experiment and the FMT experiment.** (a) Serum TG and (b) TC levels between ABX-naïve and ABX-treated mice in two sexes. (c) Serum TG and (d) TC levels among different groups in two sexes. (e) Relative Col1a1 mRNA expression of iWAT and (f) gWAT among different groups in two sexes. (g) Liver TG levels among different groups in two sexes. (h) Col1a1 protein expression of liver among different groups in two sexes. (i) Serum ALT, (j) AST and (k) AST/ALT levels among different groups in two sexes. Values are expressed as the mean ± sem. *, p<0.05; **, p<0.01; ***, p<0.001; ns, not significant. Abbreviation: M6W, ABX-naïve male mice; F6W, ABX-naïve female mice; M6W-ABX, ABX-treated male mice; F6W-ABX, ABX-treated female mice; Control-M, ABX-naïve male mice; PBS-M, ABX-treated male mice gavaged with PBS; MGM, ABX-treated male mice gavaged with male mice fecal slurry; Control-F, ABX-naïve female mice; PBS-F, ABX-treated female mice gavaged with PBS; FGF, ABX-treated female mice gavaged with female mice fecal slurry; BAT, brown adipose tissue; iWAT, inguinal white adipose tissue; gWAT, gonadal white adipose tissue; TG, triglyceride; TC, total cholesterol; ALT, alanine aminotransferasens; AST, aspartate aminotransferase; AST/ALT, the ratio of AST versus ALT.

**Supplementary Figure 4.** **Gut microbiota, lipid levels, hepatic function and glucose tolerance indicators among HFD mice groups in two sexes.** (a) PLS-DA plot of fecal-derived gut microbiota in male HFD mice. (b) Comparison of top 10 key species relative abundance among male HFD groups. (c) Comparison of top 10 key species relative abundance among female HFD groups. (d) H&E staining of BAT, iWAT and gWAT among male (up) and female (down) HFD groups. (e) Serum TG, (f) TC, (g) ALT, (h) AST and (i) AST/ALT levels among different HFD mice groups in two sexes. (j) Blood glucose and AUC curve of IPGTT in male HFD mice. (k) Blood glucose and AUC curve of IPGTT in female HFD mice. For b and c, values are expressed as the median. For e-k, values are expressed as the mean ± sem. *, p<0.05; **, p<0.01; ***, p<0.001; ns, not significant. Abbreviation: TG, triglyceride; TC, total cholesterol; ALT, alanine aminotransferasens; AST, aspartate aminotransferase; AST/ALT, the ratio of AST versus ALT; AUC, area under the curve; IPGTT, intraperitoneal glucose tolerance tests; Control-M-HFD, ABX-naïve male mice fed with HFD for four weeks; PBS-M-HFD, ABX-treated male mice gavaged with PBS fed with HFD for four weeks; MGM-HFD, ABX-treated male mice gavaged with male mice fecal slurry fed with HFD for four weeks; Control-F-HFD, ABX-naïve female mice fed with HFD for four weeks; PBS-F-HFD, ABX-treated female mice gavaged with PBS fed with HFD for four weeks; FGF-HFD, ABX-treated female mice gavaged with female mice fecal slurry fed with HFD for four weeks; BAT, brown adipose tissue; iWAT, inguinal white adipose tissue; gWAT, gonadal white adipose tissue.

**Supplementary Figure 5.** **The serum metabolites and top 10 key gut bacterial species altered by ABX and FMT intervention in standard chow diet mice.** (a) Volcano plot of metabolites in M6W-ABX vs M6W. (b) Volcano plot of metabolites in PBS-M vs Control-M. (c) Volcano plot of metabolites in MGM vs PBS-M. (d) Volcano plot of metabolites in F6W-ABX vs F6W. (e) Volcano plot of metabolites in PBS-F vs Control-F. (f) Volcano plot of metabolites in FGF vs PBS-F. (g) The relative abundance of top 10 key fecal-derived gut bacterial species in males in the ABX intervention experiment. (h) The relative abundance of top 10 key fecal-derived gut bacterial species in males in the FMT experiment. (i) The relative abundance of top 10 key fecal-derived gut bacterial species in females in the ABX intervention experiment. (j) The relative abundance of top 10 key fecal-derived gut bacterial species in females in the FMT experiment. For g-j, values are expressed as the median. *, p<0.05; **, p<0.01; ***, p<0.001; ns, not significant. Abbreviation: M6W, ABX-naïve male mice; F6W, ABX-naïve female mice; M6W-ABX, ABX-treated male mice; F6W-ABX, ABX-treated female mice; Control-M, ABX-naïve male mice; PBS-M, ABX-treated male mice gavaged with PBS; MGM, ABX-treated male mice gavaged with male mice fecal slurry; Control-F, ABX-naïve female mice; PBS-F, ABX-treated female mice gavaged with PBS; FGF, ABX-treated female mice gavaged with female mice fecal slurry.

**Supplementary Figure 6.** **The top 10 key cecal-derived gut bacterial species altered by ABX, FMT intervention in standard chow diet and HFD mice.** (a) The relative abundance of top 10 key cecal-derived gut bacterial species in males in the ABX intervention experiment. (b) The relative abundance of top 10 key cecal-derived gut bacterial species in males in the FMT intervention experiment. (c) The relative abundance of top 10 key cecal-derived gut bacterial species in females in the ABX intervention experiment. (d) The relative abundance of top 10 key cecal-derived gut bacterial species in females in the FMT intervention experiment. (e) The relative abundance of top 10 key cecal-derived gut bacterial species in males on HFD. (f) The relative abundance of top 10 key cecal-derived gut bacterial species in females on HFD. For a-f, values are expressed as the median. *, p<0.05; **, p<0.01; ***, p<0.001; ns, not significant. Abbreviation: M6W, ABX-naïve male mice; F6W, ABX-naïve female mice; M6W-ABX, ABX-treated male mice; F6W-ABX, ABX-treated female mice; Control-M, ABX-naïve male mice; PBS-M, ABX-treated male mice gavaged with PBS; MGM, ABX-treated male mice gavaged with male mice fecal slurry; Control-F, ABX-naïve female mice; PBS-F, ABX-treated female mice gavaged with PBS; FGF, ABX-treated female mice gavaged with female mice fecal slurry; Control-M-HFD, ABX-naïve male mice fed with HFD for four weeks; PBS-M-HFD, ABX-treated male mice gavaged with PBS fed with HFD for four weeks; MGM-HFD, ABX-treated male mice gavaged with male mice fecal slurry fed with HFD for four weeks; Control-F-HFD, ABX-naïve female mice fed with HFD for four weeks; PBS-F-HFD, ABX-treated female mice gavaged with PBS fed with HFD for four weeks; FGF-HFD, ABX-treated female mice gavaged with female mice fecal slurry fed with HFD for four weeks.
